# Impact of Nurse-Patient Relationship on Quality of Care and Patient Autonomy in Decision-Making

**DOI:** 10.3390/ijerph17030835

**Published:** 2020-01-29

**Authors:** Jesús Molina-Mula, Julia Gallo-Estrada

**Affiliations:** Nursing and Physiotherapy Department, Universitat de les Illes Balears, 07122 Palma, Spain; j.gallo@uib.es

**Keywords:** nurse-patient relationship, decision making, personal autonomy, quality of health care, nurse’s role

## Abstract

Background: The patient is observed to acquire a passive role and the nurse an expert role with a maternalistic attitude. This relationship among others determines the capacity for autonomy in the decision making of patients. Objectives: The aim of this study is to analyse the nurse-patient relationship and explore their implications for clinical practice, the impact on quality of care, and the decision-making capacity of patients. Design: A phenomenological qualitative study was conducted. Settings and participants: Thirteen in-depth interviews with nurses and 61,484 nursing records from internal medicine and specialties departments in a general hospital from 2015–2016. Methods: A discourse analysis and triangulation for these sources were conducted. Results: The category elaborated from nursing records was defined according to the following codes: Good Patient, Bad patient, and Social Problem. Analysis of the interviews resulted in a category defined as Patient as a passive object. Discussion: A good nurse-patient relationship reduces the days of hospital stay and improves the quality and satisfaction of both. However, in contrast, the good relationship is conditioned by the patient’s submissive role. Conclusion: An equal distribution of power allows decisions about health and disease processes to be acquired by patients, autonomously, with the advice of professionals. The nurse-patient relationship should not pursue the change in values and customs of the patient, but position the professional as a witness of the experience of the health and illness process in the patient and family.

## 1. Introduction

In general, most of the literature does not deal with behaviours and communication skills between the nurse-patient relationship and the role of both. There has been a large amount of documentation on self-care strategies, medication adherence, psychological interventions, and patient and nurse satisfaction [1,2,3,4]

Studies on the implications of this relationship in the perceived improvement of patient care are also common [5]. Many studies have focused on the exploration of nurse-patient relationships based on empirical studies [6,7,8,9]. Most of the literature refers to the experiences of nurses and are narrated in a personal tone [10].

This research focuses on how the nurse’s relationship with the patient can affect the capacity for autonomy in the latter’s decision making. Although it is a phenomenon that is affected by many other factors such as interprofessional relationships, the health institution, care models, etc.

## 2. Background

The most concurrent topics in the literature reviewed on nurse-patient relationship are the (a) role of the patient, (b) role of the nurse, and (c) type of nurse-patient relationship.

### 2.1. Role of the Patient 

A significant number of authors agree that the patient is usually considered a passive subject [11,12]. Thus, it is understood that, classically, a person who assumes a rigorously passive attitude, who does not ask or protest and who obeys all the mandates is considered a “good patient”. This conception places excessive emphasis on the authority of the professional regarding the patient [13]. 

Hallet et al. [1] associated the passive role as a form of paternalism, wherein the patient is seen as a subject who must be limited to comply with the therapeutic indications. Kleiman, Frederickson, and Lundy [14] relate the passive role with the belief of professionals that many patients, or most of them, do not have the educational and cultural levels necessary to engage in an equal professional relationship [15]. 

The reviewed works show that patients focus their relationship with the nurse on trust [16]. The patient trusts the nurse and allows her to make decisions, being treated as a minor. Many authors see the origin of this role in the Oath of Hippocrates that places a large emphasis on the power, knowledge, and virtues of the professional, without mentioning anything about the autonomy of the patient [4,17,18]. 

Henderson [19] notes that this domination over the patient causes a depersonalization and, consequently, a worsening of the relationship between the nurse and the patient [11,20].

### 2.2. Role of the Nurse 

The bibliography refers to two types of roles that are well differentiated from the nurse: The role of an expert nurse, and the role of a maternal nurse.

Most authors describe the practice of nursing as empirical [21], wherein cognitive, behavioural, integration of skills, values, and beliefs are relegated to a secondary level.

Other authors consider the expert role to be circumscribed by a collection of fragments of information only on biological aspects of the patient [22]. In contrast, Brown [23] considers an expert professional to direct his knowledge, professional experience, and clinical skills to the specific objectives of each patient. Thus, the conceptions of the expert role of the nurse are very heterogeneous.

Regarding the maternal role, authors refer to a position of the nurse that is more intimate than desirable [24,25] or is based on the concepts of adherence [26], collaboration [27], concordance [28], and compliance [29].

It is interesting to note how some studies identify the role of nurses as simple informants of treatment regimens based on the assumption that if patients understand, they will be compliant, again highlighting the maternalistic role of the nurse [3,4,17].

### 2.3. Types of Nurse-Patient Relationships

The studies analysing the type of nurse-patient relationship focus on concepts of compliance, empowerment, quality of the relationship, impotence, and power.

Compliance is defined as the will to develop and consent to the wishes of another person [29,30]. Along with the term compliance [31,32], others have been considered, including empowerment [32,33] and association [11,34]. These studies refer to the nurse-patient relationship as an interaction between both based on the patient’s autonomy in making decisions after listening to the advice of professionals.

Regarding the quality of the relationship, it appears to be a factor that influences the professional to promote patient-centred care [2]. Luker, Austin, Caress and Hallet [35] argue that this quality of the relationship directly affects the quality of care provided and is crucial for the effectiveness of the nursing practice.

Regarding the concept of impotence, the authors analyse the nurse-patient relationship according to the difficulties experienced by the nurse to observe the real needs of the patient without clinical practice guidelines or evidence-based practice support [1]. However, in addition, this impotence is generated when the patient does not receive the expected attention from professionals [36]. 

Finally, a concept present in the literature is that of power [15,37]. Cameron [31] defined it bilaterally, in which the professional develops a “reference power” in the patient, and for the patient, the professional is considered a “frame of reference”. The patient’s perception of the professional will be configured: An expert who exercises power over the decision-making process during care [24,38].

The health system has developed strategies with the aim of humanizing care and improving the quality of care. However, now the management models have not allowed a real autonomy of the patient in the decision making.

The nurse-patient relationship is one of the aspects that affects the patient’s autonomy. Analyzing the types of relationships allow us to establish new ways of understanding the decision-making capacity of patients in a clinical setting. The analysis of nursing records and nurse discourses have shown that our professional practice is not yet totally patient-centred.

A good nurse-patient relationship reduces the days of hospital stay and improves the quality and satisfaction of both. However, in contrast, although the patient’s participation in their decisions is greater, the good relationship is conditioned by the patient’s submissive role [39,40,41,42].

A poor or bad nurse-patient relationship decreases the quality of care and diminishes the patient’s autonomy. A bad patient is considered who demands a lot of information, who wishes to make his/her own decisions, sometimes, contrary to those recommended by professionals, who does not maintain a good relationship with professionals [40,41].

The aim of this study is to analyse the factors that condition the nurse-patient relationship and explore the implications for clinical practice, the impact on quality of care, and the decision-making capacity of patients.

## 3. Materials and Methods 

### 3.1. Research Design

A phenomenological qualitative study was conducted. A discourse analysis was conducted for two sources. On the one hand, through in-depth interviews with nurses, and on the other hand, nursing records about the clinical evolution of patients from internal medicine and specialty departments in a general hospital. 

This study on nurse-patient relationships belongs to a larger one that has also analyzed interprofessional relationships and the relationship of the nurse with management models.

This article analyses the patient’s autonomy in decision making and the factors that condition it, based on the criticism of the current ethical model in the clinical setting from the perspective of Foucault [43,44].

Knowing the connection between the patient’s autonomy and power’s exercise in daily practices in a hospital is essential to understand and articulate a Foucauldian ethic. For this reason, the mechanisms and procedures that are present in the exercise of strategies of normalization, homogenization, impositions, restraints, oppressions, and knowledge that determine the patient’s autonomy capacity in making decisions must be analysed [45,46,47].

The perspective of the Foucauldian ethic analyses the autonomy based on the codes that currently configure the behaviors allowed or forbidden as opposed to personal choice that opens a new possibility of understanding ethics. This analysis allows us to establish a new way of understanding the subject as an autonomous being. Foucault’s ethical proposal aims to break with the ethics based on universal principles, giving rise to a greater leadership to the patient’s self-determination [48,49].

### 3.2. Sample/Participants

Nursing records were collected throughout the year 2019 and interviews were conducted from March to December 2019.

The Department of Internal Medicine and Medical Specialties compared to the other units, has the most types of pathologies attendance and different types of professionals are present.

With respect to professionals from both services, we highlight the following characteristics: Nurses (23 regular staff and approximately 70 temps per year) working three shifts, in the morning, afternoon and night, with an average age of 33 years and a minimum experience of five years in service and nine years as a nurse.

The reasons for selecting these units are as follows: (a) These are the services with the highest occupancies in the hospital. This allows an analysis of the phenomenon in greater depth because there is greater wealth in the nurse’s relations with the patient and other professionals; (b) since there are different pathologies, this allows different types of care and therefore, greater complexity in the nurse-patient relationships; (c) these services have the highest health care burdens (data obtained from the system of Related Groups for the Diagnosis of the Health Center, which establishes the workload and distribution of necessary health personnel). In this way, the tensions and resistances that arise between the nurse and the patients can be analyzed in greater depth; and (d) these services are associated with a greater number of professionals on staff, but a lower nurse-patient ratio and a greater diversity of the specialties involved in patient care.

#### 3.2.1. Nursing Records 

Nursing observation records are documents in which nurses collect the assessments and incidences on the care and clinical evolution of the patients. These documents include all activities that the nurse performs during her work shift and are recorded in the medical histories.

In the Spanish health system, the nursing records are annotations that nurses make about the patient’s clinical evolution, as well as the care plan for these patients during a work shift. These records can be accessed by nurses of all shifts assigned to the patient, their doctors, and other professionals as assistants. Only the healthcare professional and not administrative staff.

The inclusion criteria were as follows: (a) Computerized records in the care management programme of the Can Misses Hospital in Ibiza and (b) clinical histories of patients who have been admitted to the unit for more than five days, with a minimum time considered to have generated greater discursive significance on the continuity of care.

#### 3.2.2. Interviews with Nurses

Of a total of 16 nurses in the two selected services, only 13 met the inclusion criteria. Ten semi-structured interviews were conducted, by which the level of theoretical saturation was reached.

The inclusion criteria were as follows: (a) Nurses with a minimum experience of five years, which is considered enough time to have some professional expertise in the development of nursing care; (b) nurses with a minimum stay of three years in the services under study, which is considered a sufficient duration to understand the structure and function of this unit and the professionals involved in the team; and (c) nurses who agreed to participate and signed the informed consent. 

### 3.3. Data Collection

#### 3.3.1. Nursing Records

All records of nursing observations that are reflected in the Patient’s Clinical Record are included in a computer care management tool called Gacela ©. This tool allows nursing observations to be obtained without needing to know the personal data of the same or of the professionals involved, which supposes an absolute anonymization of the data and therefore a guarantee for the participants’ right to confidentiality.

Both the nursing records and the clinical unit have been selected through intentional sampling. Initially, all records for the year 2015 were used for their chronological reading from January to December of the internal medicine unit, totaling more than 60,000.

This hardly manageable amount for a study of these characteristics raised a series of strategies for definitive selection. These strategies were as follows: (1) Select a series of key words regarding the core concepts of the study and review the literature, which by means of a sweep with the Atlas.ti program provided a first approximation of the saturation of the discourse; (2) select the months that generated the greatest amount of discourse regarding the study phenomenon. For this reason and taking into account the months that met the inclusion and exclusion criteria, the months of January, February, March, and April were selected; and (3) after selection, the data were exported to Atlas.ti and its categorization and codification.

The records were stored in a database from which the preliminary analysis has been developed. The first step of the data collection consisted of the literal reading of the records. An encrypted code was assigned to each patient and professional corresponding to the records, and later records were classified by the same registration number to ensure the inclusion and exclusion criteria.

#### 3.3.2. Interviews with Nurses

An in-depth interview technique was used via non-intrusive or direct yet comprehensive interactions within the framework of the participants, observing reality as they experience it and how they view the study phenomenon.

For the recruitment of nurses, they were contacted through the hospitalization unit and invited to participate. After accepting, the information sheet and informed consent were given. A day and time was specified with them for the realization and recording of the interview.

The entire data collection process was accompanied by the development of a field diary and dual digital recording in the presence of an observer, who collected data on metalanguage, positions, and gestures, among others. The minimum duration of the interview was 34 min, and the maximum was 68 min. After the recording was obtained, it was incorporated into the database for the study for the sole and exclusive use of the research team members. After checking the audio, the recordings were delivered to the transcriber, who had previously signed a confidentiality agreement regarding the data. To ensure data anonymity, each transcript was reviewed, and symbols were substituted for the names of people or places. From then on, every interview was assigned a hermeneutic unit by the qualitative programme ATLAS.ti version 6 (Scientific Software Development GmbH, Berlin, Germany).

### 3.4. Ethical Considerations

This research involves completely confidential data and sources from the personal data of patients to the professionals who treat them. This study was approved by the Ethics Committee for Clinical Research of the Balearic Islands and was authorized by the hospital. With respect to the recorded interviews, once the digital files were transcribed, the recordings were discarded. In addition, the study participants received information about the research and signed an informed consent document developed for this study.

We respected the legislation in Spain and the principles of the Declaration of Helsinki and other international recommendations with regard to data protection. No conflict of interest existed between participants and researchers conducting the study. 

### 3.5. Data Analysis

Discourse analysis was used to explore the factors that condition nurse-patient relationships and their impact on the quality of care and the patient’s autonomy in decision-making from the nurses’ perspectives. A discursive analysis was used to reconstruct the meaning of the text from the particular to the general. Association and interpretation along with the extraction of conclusions consisted of interpreting the transcripts and their implied significance based on Foucault’s poststructuralist theory. 

#### 3.5.1. Validity and Reliability/Rigour

The strategy of methodological rigor was carried out through a field diary in which the steps and decisions were assumed during the development of the investigation of two analysed sources, and the nurses’ records and in-depth interviews with nurses were described.

#### 3.5.2. About the Nursing Records

From the documentation technique, the nursing records were selected. This technique has contributed the narrative of the texts in a temporal order, with key arguments used to analyse the nurse-patient relationship.

With the electronic anonymized format, the records were selected according to the inclusion and exclusion criteria established in the methodological design. The following table (Table 1) presents the documental process of selection of records from the first screening performed.

In the table, a change in the number of records is highlighted that affects the inclusion and exclusion criteria expected from the month of April. This situation is due to two clearly differentiated factors. On the one hand, the criteria refer to the nurses in the service who, as of the month of April, initiated vacations periods and were replaced by temporary personnel with no minimum experience of three years in the service. In addition, nurses who acted as reinforcements of personnel with very short contracts due to workloads caused by the increase in the population in summer periods were identified as another cause of the aforementioned change.

On the other hand, in reference to the criterion of patients admitted for at least five days, from the month of April and especially during the summer months, changes in the pathologies of admission, with more acute episodes and the overload of other services, resulted in differences in internal medicine. Taken together, these findings explain why the records selected in this first phase decreased according to the established criteria and justified the methodological approach.

Using very brief and descriptive records, we decided that references to purely clinical values such as vital signs, diagnostic tests, or delegated activities would be omitted. 

Due to the characteristics of the records, these being very brief, sometimes non-existent, we tried to cover most of the records and use those that presented the greatest discursive load for a deeper discourse analysis. Despite this, the number is so high, considering that certain ways of registering provided a wealth for the analysis.

#### 3.5.3. About Interviews with Nurses

Interviews with nurses are included to both expand the information of the records and compare a written discourse of them with that obtained through an in-depth interview of the nurses about the phenomenon under study.

The interview script was elaborated based on the objectives of the study and the thematic categories that emerged during the literature review. Two interviews with nurses were scheduled in January 2019 for similar services from another hospital centre to pilot this script. From this piloting, some questions were modified due to difficulties in compression and response. In this way, the final script of the interview was established, taking into account the semi-structured interview technique in depth.

At the beginning of each interview, the sociodemographic data were collected for the participants (Table 2).

The use of various tactics such as facilitation-animation, reaffirming and repeating questions, clarification of the concepts asked, and recapitulation was required.

The transcription included a series of common elements for the representation of silence and other aspects of the discourse (Table 3).

The triangulation consisted of the delivery of four interviews transcribed to each member of the research team and three meetings for sharing the transcriptions. It began with an initial inductive analysis without pre-established categories or codes, although the topics described in the literature review and the objectives established in the study were considered as references. We then proceeded to the elaboration of certain categories that encompassed these discourses, and an approximation of the codes of each category was made according to the different discursive positions. The comments collected increased the methodological rigor of the research and provided key elements for its subsequent categorization.

Next, the refined and sensitive discourse analysis phase began. This process allowed the division of each code in different discursive positions before the phenomenon of study, as well as the configuration of a series of relationships (association, causal, and transitive) between the different codes based on the factors determined by the studies carried out in this regard according to the literature review.

All these aspects established the criteria used to assess the methodological rigor of the study. These were credibility, auditability, and transferability, in accordance with Morse [27]. 

### 3.6. Research Limitations

In relation to the particular limitations of the technique of collecting data from nursing records, it should be noted that since it is a very extensive documented source, with concise and unstructured texts, we have established a selection strategy to ensure the analysis of contextualized discourse in the clinical setting. This selection is meant to circumscribe certain months of the year and the discrimination of those registries that did not contribute, due to their grammatical structure, information relevant to the phenomenon under study.

## 4. Results

### 4.1. Nursing Records in Clinical Histories

Singular characteristics of the construction of nurses’ discourse on the clinical evolution of a patient are observed. The set of nursing registers are, for the most part, brief, unstructured, centred on clinical plots of the patient, and without connection to each other. Depending on the narrative style of the nurse, more or less punctuation marks, separations and use of capital letters appear. The structure of the discourse also demonstrates the ease or difficulty to express certain situations and the greater or lesser interest of the nurse in preparing such discourses.

The category elaborated from the discourse analysis of the registries has defined the Be Patient with the following codes: *Good Patient*, *Bad patient*, and *Social Problem* from the Nurse Perspective (Table 4). 

It is evident that the nurse prefers a submissive and passive patient. Hence, two types of patients are fundamentally conceptualized. The first is a patient as an object of care, who is described as collaborative and participatory and with a limited ability to make decisions. The other patient, the one that generates discomfort because of his attempts to make decisions about his care, leads to denial in the face of certain interventions or nonconformity for perceived attention. A small number of records describes other patients who can be characterized by their clinical and socio-familial situation and are classified as agitated or with social problems.

The definition of a “good patient” is one who collaborates and participates in what the professionals indicate, who moderately expresses their feelings and concerns, who is autonomous in basic activities such as hygiene, food and elimination, and who ultimately does not make decisions about his care, clearly providing a more comfortable role for the nurse.

A “demanding” patient, one who is difficult or uncomfortable, who denies professional imperatives and is agitated neurologically, is perceived by the nurse as a “bad patient” due to the increase in workload and tensions that arise in the relationship between both. Hence, the preference of the nurse for a patient with a passive role in care with little or no role in decision making is reinforced.

Finally, another conceptualization of a patient classified according to their degree of physical autonomy and socio-familial assessment appears, which does not provoke a negative or positive perception in the nurse but rather a maternalistic attitude in this regard.

### 4.2. Nurses Interviewed

The profile of the interviewed nurses was mostly women, aged from 29 to 54 years, with professional experience between nine to 28 years and who have developed their professional practice in internal medicine and medical specialties services for at least three years, although some have been working for up to 24 years. The employment situation of the interviewees was permanent staff of the health service, interim personnel, or on commission of services. This indicates stability in both groups, although some of them were only for a few months.

Although segmentation variables were not considered, the sociodemographic characteristics were described according to the homogeneity in the distribution of the groups, allowing the distinction of two stripes in each characteristic to analyse the differences that appear in the discourses.

Analysis of the interviews has generated a category in the patient-nurse relationship, confirming what was found in the registers and what has been defined as *Patient as a passive object of care*. 

The codes that have appeared in this category are Protective paternalism with the patient, Tensions in the relationship with the patient, Power of decision of the patient, Strategies of power of the nurse, Good patient for the nurse, and Impact of the nurse relationship-patient in care (Table 5).

It was found that for the nurse to support, empathize, advise and care for a patient, and ultimately, to develop a good relationship with him, the degree of participation in the care and attitude acquired by the patient in this relationship are decisive.

In this sense, the majority of nurses interviewed asserted that the patient must accept his illness, collaborate, and carry out all indications imposed on him by the professionals. Otherwise, there are conflicts that cause changes in the attitude of the nurse and configures the concept of the “good” or “bad” patient.

The type of nurse-patient relationship determines the making-decision of patients. Nurses with fewer years of professional experience believe that physicians should make the last decision about care. The nurse only makes decisions about basic care and, in some cases, after consulting the doctor. However, nurses with more experience recognize their influence on the decisions of the doctor.

Likewise, a preference for an obedient patient appears in the discourse of younger nurses and those with fewer years of professional experience. Having more experience is related to assuming that patient decision-making depends on the degree of participation allowed by the nurse. They recognize that the guidelines are established by the professional or the institution and must be enforced.

The nurse controls the relationship in terms of empathy and direct treatment with a paternalistic attitude, and the patient accepts this control, in these terms. This relationship is efficient, stable, and predictable if each one acquires the established role. Thus, the patient’s decision-making capacity is limited to assuming the passive role that is the object of care.

In addition, certain differences are observed in nursing discourses according to their age and professional experience. Although in both groups the relationship with the patient is based on trust, in nurses with a younger age and less experience, this trust is given when the patient values and respects the work of the nurse. In the older and more experienced nurses, the interpersonal relationship with the patient is prioritized to generate this trust.

In all the described roles, the capacity for the decision-making of the patient is limited, demonstrating a greater presence in young nurses and those with little experience. In older and more experienced nurses, there is a greater recognition that this autonomy is limited by the professional.

## 5. Discussion

This study reveals that the patient is not autonomous in making decisions about their care due to the characteristics of the nurse’s relationships with the patient, as an important factor among others. It is these elements that describe the current situation in a hospital setting and the ability of the patient to make decisions regarding the kind of care they want from the perspective of the nurse.

There continues to be a crossroads between a model of protective paternalism and an informed choice model [2,17,50,51,52,53,54]. In this study, both models are intermingled, but the dominance of the first one is observed.

It is noted in the results that the professionals are those who make decisions on the patient’s care. The records provide the clinical characteristics of the patient, but there is no consistency in terms of their identity or their decision. When reviewing the records, the reference to psychological aspects or decision making, are practically absent. Most refer to hemodynamic or purely clinical aspects: “hemodynamically stable patient” “has no fever”. While in the interviews, the decisions are based on the confidence about how the technical aspects are relegated to the background. In both sources, the records and interviews, the silence of the patient in the decision making is evidenced.

The nurse constantly justifies any decision about care, for the benefit of the patient, without leaving a space for their autonomy. Faced with a clinical situation, the professionals decide based on scientific evidence. On some occasions, but not always, they take into account the opinion of patients as revealed by Epstein and Peters [55], Nelson, Han, Fagerlin, Stefanek and Ubel [56], Sevdalis and Harvey [57] and Dowie [58]. This study dominates a restrictive conception about the decision-making of patients in our clinical setting.

Despite the dominance of the restrictive model, certain discourses emerge in nurses, which emphasize the need to involve the patient, advising on the different possible care alternatives. The nurse-patient relationships, although characterized by trust, remain fixed and marked by the roles assumed by each in that relationship, as defined by Enmanuel [59]. Therefore, the limitations of practical operationalization of a more open model in the decision making of the patients proposed by Cribb and Entwistle [50] are manifested.

### 5.1. Autonomy in the Decision-Making of the Patient According to the Relationship with the Nurse

**Figure 1:**
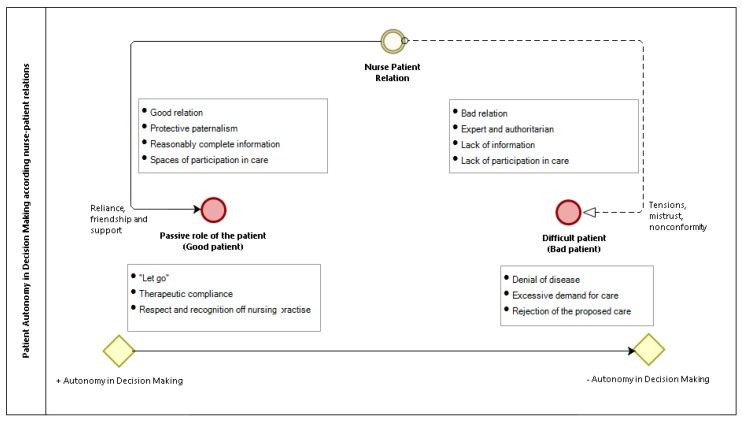
Autonomy in the decision-making of the patient according to the relationship with the nurse.

Both records and interviews confirm that the nurse prefers a submissive and passive patient who complies with the therapeutic indications (Figure 1), which would explain why the nurse focuses on patient clinical records in an impersonal way, in which the patient’s voice only refers to situations of pain or subjective perceptions.

This idea is reinforced in interview discourses, in which the nurse assumes that the patient must trust the professional fully and let go. Thus, when the patient enters a hospitalization unit, figuratively, he/she becomes the property of the professionals and is therefore dominated by them, as indicated by Arrollo Arellano [13].

The “good patient” is conditioned by the degree of submission that the patient acquires in the relationship with the nurse. The results of the present study also reveal that to be considered a “good patient”, characteristics such as accepting the disease and not continuing denial, not requiring a lot of information, and not requiring attention, must be present assuming the rules and norms of the institution, that the work of the nurse must be valued and that a collaborative and participatory role is observed in the imposed care.

### 5.2. Perception about the Patient

The results show that the nurse is immersed in a dialectical duality to define a good or bad relationship with the patient. This definition inevitably passes by cataloguing the patient as “good” or “bad”. While the profile of a “good patient” is not clear. In general, a “good patient” from the nurse’s perspective is defined by a patient without identity, trusting, without information, and grateful, i.e., a submissive patient, passive object of care, and without decision power. Important difficulties are observed to refer to the characteristics of a “bad patient” depending on the role and attitude acquired by the nurse in the relationship.

The common denomination in both records and interviews is that of the “demanding” or “very demanding” patient, which curiously appears in records as a sign of alarm among professionals to initiate a series of preventive strategies in the relationship with the patient. Demanding patients are those who, in general, increase the workload of the nurse, with respect to what she considers should be dedicated to each patient according to their pathology. Such patients do not obey the orders of professionals, refuse treatment and care, and demand a lot of information.

A good relationship with the patient, in contrast, is accompanied by mutual trust, cordiality, closeness, resolution of doubts, counselling, empathy and even friendship, as widely contextualized in other studies [4,7,21]. The bad relationship poses a positioning of the nurse as an expert in care, accompanied by a distancing towards the patient, with continuous conflicts, scarce communication, a decrease in time dedicated to the patient and their concerns, and a relationship centred purely on technical aspects.

### 5.3. Nurse-Patient Relationship

There is a direct impact on the quality of care depending on the type of relationship with the patient. The nurse recognizes that a good relationship improves the quality and healing results in the patient, as previously described in the study by Ramos [60].

Each of these patient conceptions carries with it an associated role and attitude of the nurse. It is evident in the results of records that before a patient refuses, the nurse acquires an expert role that entails persuasion and coercion to achieve the indicated healing objectives [15,61]. In the interviews, the nurse appears as an expert in the delegated activities from physicians, in association with technical skills and clinical knowledge about the pathologies. An interesting dichotomy between records and interviews emerges in this sense. 

While the texts of the interviews are more valued communication skills and relegates to the background techniques, the records show that most of them only refer to purely technical aspects. Nurses in verbal discourse, bet more on aspects of empathy, understanding, and emotional approach to the patient. However, the records, for the most part, express clinical values, hemodynamic stability, etc.

Additionally, although this role can be confused with empathy, it ceases to be so when a continuous manifestation of care that annuls the patient’s autonomy for their benefit and survival appears in the interviews.

The patient perceives the nurse as one who makes decisions about his/her care, becoming a reference of imposed power, as indicated in the study by Buchmann [29]. In this dominating-dominated binomial, the patient acts as dominated, and the nurse manipulates the power, alleging a protectionist attitude towards the weakest part using strategies of power such as manipulation, persuasion, and coercion and therefore exercising a role, paternalistic or as an expert, of domination.

This situation causes a homogenization of the patient that threatens their identity and hinders the individuality and uniqueness of the people.

The present data indicate that the free and autonomous behaviour of the patient in the clinical setting is, insofar as the health institution and professionals are involved, especially the nurse, inclusive of the patient’s capacity for decision-making competencies.

The factors analysed in this work show that the patient’s decision-making power over their care must be explained through the nurse-patient relationship.

## 6. Conclusions 

The passive role of the patient acquires its maximum expression in hospitalization units, in which the context is assumed to lack autonomy to participate in their care and decisions regarding treatment. 

A quasi-parental role of the patient’s professional and quasi-infant provides a mixture of beneficence and power. The nurse aims to benefit the patient, justifying their paternalistic role, and nullifying the decision-making capacity of the patient. 

An equal distribution of power allows decisions about health and disease processes to be acquired by patients, autonomously, with the advice of professionals. The nurse-patient relationship should not pursue the change in values and customs of the patient, but position the professional as a witness of the experience of the health and illness process in the patient and family.

The results of this research show the use of language as a tool of power, exercised through orders to patients. These orders are based on persuasion before those who resist the impositions of professionals. The language used is based on a sealed communication and the use of terms of affectionate content.

This research allows the following: (1) Guiding health organization managers to establish strategies that increase the autonomy of patients in decision-making, (2) modifying those aspects that limit the decision-making power of patients, and (3) implementing new ways to understand the management of health organizations that favour the relationship between members of the healthcare team and the interaction of professionals with the patient, focusing these strategies on the decision-making capacity of the patient in the clinical setting.

**Contribution of the Paper**

*What is already known about the topic?*
  - Analysing the type of relationship of the nurse with the patient allows establishing strategies to improve the quality of care and the degree of satisfaction of both.
  - Knowing the impact of the nurse-patient relationship on the autonomy of patients allows to increase their capacity in decision-making.
  - Evidence of limitations in nurse-patient relationships leads to a change in the patient-centred healthcare model.
*What this paper adds?*
  - Nursing records show an absence of the patient’s autonomy in decisions making about their care. In the interviews, it is evidenced in nurses of more experience and age, a greater degree of participation of the patients.
  - Nurses prefer a submissive patient, who assumes care without discussion and respecting the work of the nurse.
  - The patient is labelled, both in the nursing records and in the interviews, as a good or bad patient according to the relationship with the nurse.
*How should the findings be used to influence policy/practice/research/education?*
  - Implementing new ways to understand the management of health organizations that favour the relationship between members of the healthcare team with the patient, improve the decision-making capacity of the patient in the clinical setting.
  - Promoting a good relationship between the nurse and the patient, based on real respect for their decisions, would lead to a less tense in the nurse practice and with fewer limitations in communication and autonomy of the patient.
  - Until now, the nursing records in the different studies had not provided information on the quality of care and patient autonomy. Although these are a complex source of information, its analysis can provide great richness in the written discourse of nurses.
  - According to the results obtained, the educational model of the nurses should be modified, directed more towards a patient-centered model.

## Figures and Tables

**Table 1:** Nursing record selection process.

N Records	Months	Nursing Records in Inclusion Criteria		Nursing Records in Exclusion Criteria		Total Selected in Phase I
		Experience > 3 years	Hospitalization > 5 days	Experience < 3 years	Hospitalization < 5 days	
9103	January 2015	9103	9103	0	0	9103
8037	February 2015	8037	8037	0	0	8037
8820	March 2015	8820	8820	0	0	8820
8821	April 2015	8821	8821	0	0	8821
7906	May 2015	3912	3912	3221 *	773 **	3912
11744	June 2015	856	856	7233 *	3655 **	856
13929	July 2015	774	774	4899 *	8256 **	774
11192	August 2015	308	308	1006 *	9878 **	308
9962	September 2015	2521	2521	5623 *	1235 **	2521
10996	October 2015	6987	6987	3210 *	789 **	6987
9379	November 2015	7036	7036	1211 *	566 **	7036
11204	December 2015	4309	4309	6253 *	321 **	4309
Subtotal		61.484		32.656	25.473	
Total						61484

* Due to the vacations of the fixed staff of the internal medicine service, the criterion of minimum of experience in the service of three years was not covered. ** Due to the summer period, patients did not stay in the service for more than five days, when they entered internal medicine due to acute pathologies.

**Table 2:** Sociodemographic characteristics of the interviewees.

Nurse	Age	Unit	Professional Experience in Years	Years in Units	Labour Situation
E1	37	EME *	11	5	Permanent staff
E2	29	EME	9	6	Eventual staff
E3	34	MIR **	13	5	Eventual staff
E4	50	EME	28	19	Permanent staff
E5	54	EME	17	24	Permanent staff
E6	39	EME	18	6	Permanent staff
E7	35	MIR	12	3	Eventual staff
E8	37	MIR	10	3	Eventual staff
E9	45	MIR	12	7	Eventual staff
E10	31	MIR	9	5	Eventual staff
E11	37	MIR	9	5	Eventual staff
E12	28	EME	7	3	Eventual staff
E13	31	MIR	9	5	Eventual staff

* Medical specialties unit. ** Internal medicine unit.

**Table 3:** Transcription techniques used.

Element	Transcription
Silence > 3 s	(Silence)
Silence < 3 s	
Laugh	(Laugh)
Laughter interviewer, interviewee, and observer	(All laugh)
Tails	Literal transcription
Doubtful attitude	(doubt)

**Table 4:** Category, codes, and definitions of nursing records about clinical evolutions of patients.

Category: Be Patient		
Code	Definition	Nursing Records
Good patient from nurse’s perspective	In an impersonal way, clinical states, vital signs and pathologies are recorded, wherein the identity of the patient disappears. The nurse gives importance to purely clinical aspects and, in some cases, to moods, as long as they relevantly impact the patient’s well-being.	*Sv (urinary catheter) carrier, peripheral venous, and oxygen therapy.* *Patient … a little anxious and nervous, stable constants, peripheral venous (18 msi (left upper arm)).* *Maintains stable constants the rest of the afternoon.* *Upon arrival at the plant: Arterial hypertension, norm-cardiac and maintenance of good saturation, conscious and oriented. Autonomous patient for basic activities of daily life.* *Not demanding and very consistent.*
	The patient’s voice appears as a mere expression of pain or subjective perceptions about clinical processes, which provide information to the nurse about how he/she feels or is concerned. This attitude is not related to the fact that the patient acquires real autonomy in the care. The nurse calls the patient a collaborator and participant, as long as he respects and assumes the marked therapeutic indications.	*Patient very collaborative and participative in her care.* *Asymptomatic. Collaborating patient.* *She goes to the shower to showers self very well.* *Patient very cooperative and committed to his/her TTº (treatment).*
Bad patient from nurse’s perspective	Nurse prefers what she calls a clean field, without a patient who causes problems. The dominant term coined by the nurse to refer to this type of patients is “demanding” or, with more emphasis, “very demanding”. This would be the first indication that alerts the nurse that these patients will require more attention, in terms of workload, and that there is thus a need to understand the negative aspects. These records may be interpreted as warnings to other nurses, so that they initiate preventive coping strategies such as patience, assertiveness or any other skills that might be useful to them, including the use of pharmacological measures of containment. It has been observed that cataloguing a patient as “demanding” indicates a hidden discourse, which seems to be understood by other professionals, since there is no reference in the records to the type of demands that arise. It can only be verified that patients are “demanding” because they continuously access the nursing control, require certain care, and request too much information when the demands are considered of little importance, especially during the night shift.	*Patient very negative to the treatment that is being given.* *Patient is very demanding and continually asks for removal of the nasogastric probe.* *At the beginning of the night very demanding, so, it was deemed necessary to change the roommate for his rest of the night.* *Patient asking for sleeping pill, demanding, asking for things but not disoriented …* *Calls constantly to say that it is uncomfortable, that we do not turn off the light, that the room is disorderly, that we incorporate it again, that he has swollen legs, … Attended to each time and given warning.* *Call uninterrupted throughout the night.*
	The next warning sign is a patient who becomes not only “demanding” but also difficult and even annoying. This more radical perception is confirmed through records of the patient’s denials of treatments and imposition of nursing care. The situations are described in which the nurse and doctor use strategies of persuasion and/or coercion with the objective that the patient follows the orders of the professionals. The dominant need to carry out these imperatives is justified by clinical criteria, patient well-being and survival. In cases in which patients disagree about the care provided, a range of tensions open up with the nurse and the other involved professionals. Thus, an attitude of dialogue with the patient is evidenced by the nurse to resolve such situations of stress, particularly in the case of failure, for which the recourse is containment measures with the intervention of the doctor.	*I am going to administer paracetamol according to the guidelines, and the patient refuses. I comment to the guardian doctor who returns to speak with the patient, who again consents.* *I tell the patient that it is necessary to channel a new venous line … the patient refuses to do so and requires me to recanalize it and heal the venous line. By telling him that I will not act as he tells me, since I do not consider it prudent, the venous line is started. The patient tells me that I should take the venous line "the first time". I comment with my partner who offers to channel that way to soften the situation.* *I am surprised that after administering the sprays he was going to administer the Salbutamol and Atrovent inhalers he has in his desk drawer on his own, and I inform him that he will ONLY be administered what we give him* *The patient refers on several occasions that she has not been given the medication … I mention that in the nursing folder, both are administered. I show him the signatures because he does not believe it. He reproaches me on several occasions that we have changed the medication schedule … I comment with the GD on all the demands and demands of the patient. He insists that the doctor come and finally calls the physician.* *The patient does not obey the treatment prescribed by the doctor, nor does he pay attention to the nursing advice.*
	There appears a third concept of patients that is perceived by the nurse as an increase in their workload and that requires a lot of care. This is a patient who is qualified as agitated or disoriented with neurological alterations. In this case, the agitation usually involves the application of pharmacological treatments and mechanical restraint. The use of these measures is justified as patient safety and the avoidance of traction of sanitary devices, especially invasive ones. In extreme situations, the patient is isolated in a room.	*In the morning, the patient escapes through the emergency door, and security is called, and the patient is found in the cafeteria bathroom. It is necessary to mechanically restrain the patient in the room.* *Uneasy patient in the early afternoon, becomes aggressive, and does not want to go to the room, requiring staff reinforcement to put him in bed. I speak with the doctor on duty, who decides to isolate the patient because of a social problem.* *Uneasy patient in the morning, agitated and aggressive, requiring the help of Dr. XX to administer 1 ampoule of Haloperidol IM, which is effective.* *Accurate mechanical restraint because the patient was pulling the bed.*
Patients with social problems * from the nurse’s perspective	The dependent patient refers to one who has a loss of autonomy for carrying out activities of daily living due to mobility problems or in situations such as dementia, respiratory problems, and very reduced levels of consciousness.	Inspiring by mouth, I tell him to perform breathing exercises by inhaling through the nose and exhaling through the mouth, although the patient fails to perform them. I try to sit him in the chair, but he does not have any strength in his legs.
	The patient catalogued as a social problem is recorded many times in relation to the absence of a social-family network. These patients are sometimes considered problematic and require extraordinary measures such as confinement to isolated rooms due to discomfort to other patients, or transfer to residences.	No family or friends, pending social services. Social problem. According to the emergency report, the woman remains hospitalized because it seems to be a social problem, she lives alone and her few relatives have difficulties related to the care of the patient.

* people who do not have a supportive social support or live in a situation of risk of poverty.

**Table 5:** Category, codes, and definitions in interviews about patient-nurse relationship.

Category: Patient as a Passive Object of Care		
Code	Definition	Interviews
Protective paternalism with the patient	It is noted that the nurse acts as a consultant to the patient, taking the doctor as a reference. The nurse bases her relationship with patients on trust and friendship to calm their anxieties and worries during the hospital stay. The nurse prefers a relationship, in the absence of conflicts with the patient, providing security and using a basic language, low cultural level, and understandable. This is directly related to a protective paternalistic attitude and the consideration that the patient does not have sufficient knowledge to understand medical language.	I1: “I think my relationship with the patient is good because I try to be there as much as possible and with the family so I can provide them the option to talk to me about anything they want, if they have any doubts, I try to solve all the doubts they have. And if I do not know how to refer them to another […] I think that more as a consultant because I´m not an expert. I always believe that the most expert person is the physician, so I am like an aide” I3: “Our role is to advise and provide confidence to be able to be calm and resolve all, (…) and well, to provide their peace while they are in the hospital, which creates numerous anxieties” I10: “[…] If you are explaining anything or whatever it is he can understand (…) at his level, you must speak at the level that he understands”
	Empathy appears in discourse as a fundamental aspect of the relationship with the patient. The nurse believes that having a good relationship should involve understanding the patient at all times. When that level of understanding is reached, the relationship improves. Younger and less experienced nurses do not recognize the limitations in regard to empathizing with the patient. Collaboration in care is considered sufficient for the patient to exercise his/her power of decision. In the case of nurses with more professional experience, greater sensitivity is indicated by the autonomy of the patient, trying to adapt the information provided to them so that they decide on some strategies for their care.	*I2: “Have empathy. For me, this is fundamental. I think that in order to have a good relationship with the patient, I have to understand… But as soon as I understand them, as soon as I start talking to them, I understand them a bit; and that’s when the relationship always improves”* *I3: “Well, an open, cordial, close and empathetic relationship … Yes, yes, it’s friendly, and it allows the possibility to understand the patient as much as possible”*
Tensions in nurse-patient relationships	It dominates a discourse where the nurse attributes tensions to the patient’s attitude towards illness or discontent or denial care provided. The lack of acceptance by the patient about his/her illness is the main source of conflict and is increased in the presence of an aggressive attitude of the patient or lack of respect for the nurse practice. This statement is what occurs in most speeches from the perspective of the nurse, being the cause of the lack of acceptance.	*I3: “…(…) she asked us for pyjamas, and we told her that we were busy (…) she placed herself in the middle of the hall and told us that we were uncool and well, we told her we did not refuse but, we just said wait a little because we are busy with another patient”* *I6: “The aggressiveness that the patient may have. That is, that above all they have an illness, which may lead to verbal or physical aggression towards you, so you try to perform in good faith. So, what I do not do, now I filter it more, but I do not like when they shout at me. Then, bad education or the one who shouts at me takes it fatally. Go, like (laughs) but when you stop screaming I’ll be back, and I’m leaving … I mean, you’re sick, but you’re not disabled”*
	In the face of conflict situations, a minority sector considers a patient’s resistant attitude to the imposed care to become a challenge, so that a series of skills must be displayed to maintain a good relationship with him. There is consensus that the patient’s denial of the proposed care generates a series of strategies of persuasion and coercion and consequently a clear limitation in the patient’s autonomy to make decisions.	*I4: “These patients, the ones that make it difficult to work, are like a challenge, and we have to be even better”* *I8: “For example, he refuses to do a test (…) then you insist and sometimes he screams at you, (…) you are seeing that it is important for him and you insist”* *I13: “And it is a test that he or she needs and refuses to do, and you have to be convinced and then of course, at one time, another, another and another because if there is a conflict in the end, you perform badly, do you not? You no longer work at ease like you want because he does not allow himself to try to solve his problem”*
	Older nurses with more professional experience not only refer to the attitude of patients as causes of stress but also consider the nurse to be responsible for the conflict due a lack of communication skills, especially empathy.	*I2: “When I perform poorly with patients, it is when I do not understand them. For patients like these, I say, what’s wrong? What is going on in his head to be pissed, angry …? Then, the relationship is already bad”*
Patient decision power	There is evidence, on the one hand, of a passive role of the patient as an object of care and, on the other hand, an active role that at its extreme is considered a difficult patient. These roles determine the ability of patients to make decisions. A patient who trusts the nurse and therefore does not make decisions and "lets go" defines the passive role. It is noted that once the patient enters the hospital, he is considered to be the property of the institution and professionals. It is expected of him to assume the rules and norms and to take care of taxes, without considering previous information. Therefore, the patient’s autonomy capacity, in this case, is limited to following the recommendations of the professionals and the institution. This limitation increases according to the low level of physical autonomy and age, and this dependence is confused with the ability to make decisions. It is observed that, to provide some information to the patient about what will be done to him, it is sufficient to cover the needs of autonomy. In younger and less experienced nurses, there is a resistance to provide information to the patient, and if it occurs, it reduces workloads. In contrast, in older nurses and more professional experience, the need to report is present in almost all cases.	I2: “I believe that patients are left to carry a lot for us. They do not assume much prominence in their care, I think. They get carried away by us … everything we tell them is what they believe. If we say that the pain comes from there, they believe it and it is already…” I4: “I cannot say that they make the decisions because we are always following the guidelines, although I think we have to make them part of why we do this or that”
	The patient who does not acquire the role of submission and performs different activities marked by the institution or those considered by the professionals should be assumed by the patient, configures a pro-active and emergent role. The nurse qualifies as a demanding patient. Demanding patients are patients who refuse to perform certain tests, refuse treatment or make their own decisions, and demand too much information. This type of patient is perceived by the nurse as a bad patient who does not accept their situation. In contrast, there is a sector of nurses who try to provide a more active role for the patient, as long as it does not generate conflicts or strongly deny the proposed care.	I1: “There are patients who, for whatever reason, are more difficult to treat or believe that they are not sick and in fact are then because they work with more difficulty because they do not want the treatment that they are getting, or they do not understand why they are receiving it” I2: “It is not the same going to heal a patient who makes it easy for you, who is nice, who understands you, who helps you compared with another patient who is not, who is moving, who is talking on the phone while you are healing…” I4: “There are people who do not get much care because they seem to regress to babies…”
	Finally, it is emphasized that the non-acceptance of the disease causes in the patient non-conformist attitudes towards care. This attitude is sometimes perceived by the nurse as aggression. In these cases, the patient is qualified as difficult or uncomfortable with an attitude of disrespect, increasing the workload of the nurse.	I7: “He does not understand that there are thirty-one more patients out there, right? Then, he only understands that he has been uncomfortable for ten minutes, and sometimes you get stressed and you get there. He responds that you do not accept what I say because you have not been standing with your arms crossed doing nothing… and you can answer wrongly. And of course, that happens”
Nurse’s power strategies	Three roles of the nurse with respect to the relationship with the patient stand out and determine the decision-making power of the patient. In the first place, there is evidence of a marked maternalistic attitude of the nurse. This maternalism, or protective paternalism, highlights the limitations of knowledge and skills of nurses in clinical situations, which are replaced by humane treatment with the patient, which is especially prominent in older nurses and those with more professional experience. These nurses are more comfortable being a support for the patient, basing their relationship on trust, friendship, and closeness. The nurse assumes that the patient considers her as the professional who can express her feelings. Care is defined as an art, and constant communication with the patient about issues unrelated to their illness is defended.	*I1: “An I expert I am not (…) Well I think he sees me as a point of support for … Like him, when he is here in the hospital, he is sick because he must rely on someone to help him solve his doubts … and you give the patient some confidence because if they do not have that trust, they will not talk to you and they will not feel well”* *I4: “I am very affectionate, I like caring a lot and also placing a little distance … I try to treat the patient with respect and to not treat them as silly, sick, sick people who seem to understand nothing and I start talking to everyone about You who also … you know? And then with time, well, I do not know, with some people evidently, because they become better, others become worse…”* *I8: “Throughout the duration, the longer period of time, for example the patient, is admitted, it is a perception of mine that he experiences a trust more as friendship and does not have as much need for you … I feel comfortable, being, therefore affectionate when you have to be, being a professional when you have to be”*
	Second, an attitude of an expert nurse is observed only when activities delegated by the doctor are assumed. This role is associated with technical skills and clinical knowledge and is determined by pre-established protocols to ensure the adequacy of the provided care. This group of nurses believes that the patient does not have the capacity to make decisions because they do not know the scientific evidence, so the information is reduced to banal issues. However, in addition, these nurses prioritize techniques on any other aspect.	*I4: “There are already some protocols, but sometimes you have to change them; we are doing all the same …”* *I6: “Let’s see if he understands that you are there, that you are a professional, that you are the nurse, yes, but that you are also the person that at any given moment can solve any doubt that he has … you introduce yourself and you already are neither the young lady, nor the lady, nor the nurse, you are so-and-so, that you are such and that you are working this turn, then he has reference, he or she, well”*
	The nurse sees herself as the physician’s link to the patient, and although it is an assumed role, situations are described in which the lack of communication between professionals provokes tensions with the patient. It is noted that the nurse defends plots of professional autonomy and tries to prevent the patient from seeing her as an assistant to the doctor. The perception that as a nurse you have a lack of professional autonomy, can cause tense interprofessional relationships, which affects the ability to make decisions to patients, due to lack of information or reproaches to the work of other professionals.	*I1: “The doctor is there five minutes a day, I’m there for a whole shift, of course, and the patients do not say practically anything with the doctor, and with us they can talk… Then, I can get some information that I can relay to the doctor the next day or leave written on the computer, which the doctor can read and use to mitigate the patient’s doubts”* *I7: “…because sometimes the patient knows things that we do not know, and we are unprofessional because sometimes I enter a room and the patient says is going to do this test, and I did not know and, of course, you are very wrong”*
Be a good patient from the nurse’s perspective	Being a "good patient" from the nurse’s perspective is defined by a patient without identity, trusting, without information, and grateful, i.e., a submissive patient, passive object of care, and without decision power.	I2: “The patient who assumes a lot of his illness. There are patients who assume it, but there are people who are in continuous denial, and I think those are the bad ones. For me” I2: “Patients who want too much care or who feel too dependent or who believe, as we say, they are in a hotel … Come on, we have to be for them constantly, I think that is when I have more difficulties with patients, when they demand too much attention” I9: “…The one who helps, the one who has, just as I want to have empathy with them, the one who has empathy also with the nurse, the one who values our work, the one who… those who have patience with us”
	It is evident that the nurse prefers a patient who follows the guidelines and who does not ask for many explanations about the provided care. In brief, the patient does not carry out their own initiatives.	I5: “…the patients are now no longer like they used to be, they want explanations for everything. They want you to explain everything you are going to do to them, what you are going to do to them, are they always waiting when you enter first, what are you going to do to me?” I12: “We want a field too clean to be able to work, right? Without problems …”
Impact of the nurse-patient relationship in care	It is observed that the good relationship with the patient improves general care. The improvement is the key of more dedication, confidence, security, empathy, and assertiveness. It is also emphasized that with a good relationship, the patient is calm and experiences an improved emotional state and that better healing results are obtained.	I2: “You go to a patient who gets along well or who understands you and all that and you help him with desire… Well, you treat him the same, do you not? (Laughs) but you go as more predisposed, you spend more time, you listen to him more, you spend more time with him and in the end, and in the end, these patients are the ones that receive more attention than those who are demanding” I7: “If you have a good relationship and have provided confidence to the patient, kindness and he feels safe, the night is a good time”
	It is noted that a poor relationship with the patient causes poor communication, which is diminished by the patient’s concerns and care, less time spent, and patient dissatisfaction. Although it is recognized that the technical aspect of care is the same, the interaction with the patient changes.	I2: “If the relationship is bad, I think the care is not the same. It’s not bad, but you do not take care of it because a bad thing does receive as much care or you do not pay as much attention” I8: “That a patient who refuses everything, a patient who does not want what you propose, a patient who you then have to change the care, and maybe they are already undergoing another kind of care”
	A discourse of professional excellence emerges, in which the nurse refuses to associate a bad relationship with the patient with collateral effects on care.	I4: “I try to be exquisite in my care, and whether I have a bad relationship or whether it is good, as if it is … No, no, no, no. In contrast, sometimes I say that these patients, the ones that make it difficult to perform your job, like a challenge, and we have to be even better”
